# Genetic diversity of soybean dwarf virus in two regions of mainland Australia

**DOI:** 10.1007/s00705-024-06142-z

**Published:** 2024-10-08

**Authors:** B. S. Congdon, M. Sharman, M. A. Kehoe

**Affiliations:** 1 Primary Industry Development, Department of Primary Industries and Regional Development, 3 Baron-Hay Court, Kensington, Western Australia 6151 Australia; 2 Ecosciences Precinct, Queensland Department of Agriculture and Fisheries, GPO Box 46, Brisbane, Queensland 4001 Australia; 3 Biosecurity and Sustainability, Department of Primary Industries and Regional Development, 3 Baron-Hay Court, Kensington, Western Australia 6151 Australia

## Abstract

Soybean dwarf virus (SbDV; family *Tombusviridae*, genus *Luteovirus*, species *Luteovirus glycinis*) is an RNA plant virus that is transmitted solely by aphids in a persistent, circulative and non-propagative manner. SbDV causes significant losses in cultivated Fabaceae, especially in subterranean clover (*Trifolium subterraneum*) pastures of mainland Australia. SbDV isolates are classified into four phenotypically distinguishable strains: YP, YS, DP, and DS. Y and D strains differ primarily in their host range, and P and S strains in their primary vector species. Genetically, Y and D strains separate into two clades in every genomic region except for the N-terminal region of the readthrough domain (N-RTD), in which P and S strains separate. SbDV diversity in Australia has yet to be investigated, so in this study, 41 isolates were collected from six different host species across two production regions of Australia: the south coast of Western Australia (‘south-west’) and northern New South Wales/southern Queensland (‘north-east’). A near-complete genome sequence of each isolate was obtained, and together with all 50 whole-genome sequences available in the GenBank database, underwent phylogenetic analysis of the whole genome nt and the N-RTD aa sequences. At the whole-genome level, the isolates separated into D and Y clades. At the N-RTD level, most of the isolates separated into P and S clades. All south-west isolates and 11 of the 31 north-east isolates were in the Y clade, and the remaining 20 north-east isolates were in the D clade. Except for one isolate that fell outside the P and S clades, all south-west and north-east isolates were in the P clade, suggesting that they are transmitted by *Acyrthosiphon pisum* and *Myzus persicae*. Available biological data largely supported the phenotypic inferences made from the phylogenetic analysis, suggesting that genetic data can provide critical epidemiological insights, provided that sufficient biological data have been collected.

**Supplementary Information:**

The online version contains supplementary material available at 10.1007/s00705-024-06142-z.

## Introduction

Soybean dwarf virus (SbDV), currently classified as a member of the species *Luteovirus glycinis* in the genus *Luteovirus* of the family *Tombusviridae* [54], primarily infects members of the family Fabaceae and is transmitted by aphids in a persistent, circulative, and non-propagative manner [48]. SbDV causes serious disease in economically important grain and pasture legumes worldwide. In Australia, SbDV frequently causes leaf-reddening, severe stunting, and, occasionally, pasture collapse of subterranean clover (*Trifolium subterraneum*) [20, 29, 30, 33], which is an integral component of the pasture feed base of Australia’s $12.3 billion wool, dairy, and red meat production industries [39]. The most recent SbDV epidemic occurred on the south coast of south-west Western Australia (WA) in 2017 [44]. SbDV also infects many other important pasture legumes, including other clover species (*Trifolium* sp.), annual medics (*Medicago* spp.), French serradella (*Ornithopus sativus*), and biserrula *(Biserrula pelecinus*) without causing obvious disease [6, 28]. The importance and risk of SbDV to Australia’s $2 to 3 billion grain legume industry is less well understood, but the virus can cause severe disease in key species such as chickpea (*Cicer arietinum*), field pea (*Pisum sativum*), faba bean (*Vicia faba*), and lentil (*Lens culinaris*) [6, 36]. In the 2013 season in northern New South Wales (NSW), SbDV was responsible for >75% of the virus-infected chickpea plants [46]. In that season, the incidence of virus infection was generally less than 5%, but it was as high as 30-50% in several crops, suggesting that, in some seasons, SbDV may be a significant contributor to disease in grain legumes.

SbDV isolates are categorised into four strains: YP, YS, DP, and DS, distinguishable by epidemiologically important phenotypes. Yellowing (Y) and dwarfing (D) strains were initially divided based on their symptom expression in soybean (*Glycine max*) [48], and further research showed that they had different host ranges; only Y strain isolates infected white clover (*T. repens*), albus lupin (*Lupinus albus*), and common bean (*Phaseolus vulgaris*), and only D strain isolates infected red clover (*T. pratense*) [7, 21, 40, 49]. However, there is evidence that some host range indicators are not strict. For example, eastern USA D strain isolates can infect white clover [45]. Several other species or cultivars may also be strain-specific hosts or differ in susceptibility and sensitivity to different strains [7, 28]. P (pisum) strains are transmitted most efficiently by *Acyrthosiphon pisum* Harris (pea aphid) [3, 33, 55], and S (solani) strains are transmitted most efficiently by *Aulacorthum solani* Kaltenbach (foxglove aphid) [50]. *Myzus persicae *Sulzer (green peach aphid) and *Aphis craccivora* Koch (cowpea aphid) also transmit P strain isolates [6, 8, 17, 45], and several other vector species have possible virus strain  specificity [8, 19, 21, 22, 28, 45, 55].

SbDV isolates have a ~5.7- to 5.9-kb positive-sense RNA genome containing five open reading frames (ORFs), some of which overlap [32]. Y and D strain isolates form separate clades when analysed at almost every region of the genome [45, 47, 50]. A recent study identified three Y subclades and two D subclades when analysing a global phylogeny of complete SbDV genome sequences [47]. Isolates form P and S strain clades when analysing the N-terminal region of the readthrough domain (N-RTD, encoded by ORF5), which plays a key role in  aphid vector transmission and specificity [47, 51]. Stone et al. [47] found an N-RTD recombinant (MD2-Y) with a P strain phenotype and thus identified 12 amino acid (aa) positions that could determine vector specificity. Furthermore, they found that the majority of SbDV sequences fall into the P clade, suggesting that *Ac. pisum*-transmitted strains are the most widespread globally.

By using the relationship between genotype and phenotype, Stone et al. [47] analysed sequences obtained from 17 eastern USA field isolates to help assess the present and future risk of SbDV to USA soybean production. There has been no substantial phylogenetic analysis of Australian SbDV isolates undertaken to understand viral diversity in production regions impacted by SbDV. In the phylogeny reported by Stone et al. [47], two previously sequenced isolates from WA and one from NSW fell into the Y and P clades. SbDV isolate Tas-1 from the island state of Tasmania has been included in two genetic studies and on each occasion fell into the Y and S clades [47, 51], which is supported by the available phenotype data for this and other Tasmanian isolates, suggesting that it is a common strain in this region [19, 25, 28]. The few studies to have examined the phenotype of mainland Australian SbDV isolates also suggest that multiple strains are present. The first Australian report of SbDV in Victoria in 1970 included an isolate that was transmitted by *Au. solani* but not by *M. persicae*, suggesting that it was an S strain isolate [33]. However, it is unclear whether the isolate was a D or Y strain, as it was able to infect red clover, white clover, and common bean. Helms et al. [19] tested two isolates from south-east NSW and isolate Tas-1, which were transmitted by *Au. solani* but not *Ac. pisum* or *M. persicae*. One isolate (WA-8) obtained during a severe epidemic in subterranean clover pastures growing on the south coast of WA was transmitted by *A. pisum* at high efficiency and by *M. persicae* at lower efficiency and infected white clover, common bean, and albus lupin, but not red clover, suggesting it was a YP strain. SbDV has been found infecting white clover in WA, South Australia, NSW, Victoria, and Tasmania, suggesting that Y strain isolates are prevalent in southern winter-rainfall-dominant locations [37, 38, 41]. Therefore, based on the genetic and biological evidence available to date, it is likely that at least YS and YP strains are present in mainland Australia. In this study, we sequenced 41 SbDV isolates collected from 2013 to 2022 from various grain and pasture legume species growing in two geographically distinct production regions of Australia, expanding our understanding of SbDV genetic diversity in mainland Australia. We then performed phylogenetic analysis of these sequences together with all 50 complete or near-complete SbDV genome sequences available in the GenBank database. Using available phenotype data and the relationship between SbDV genotype and phenotype, we provide the first assessment of SbDV diversity in Australia.

## Materials and methods

### Isolate collection

**Fig. 1:**
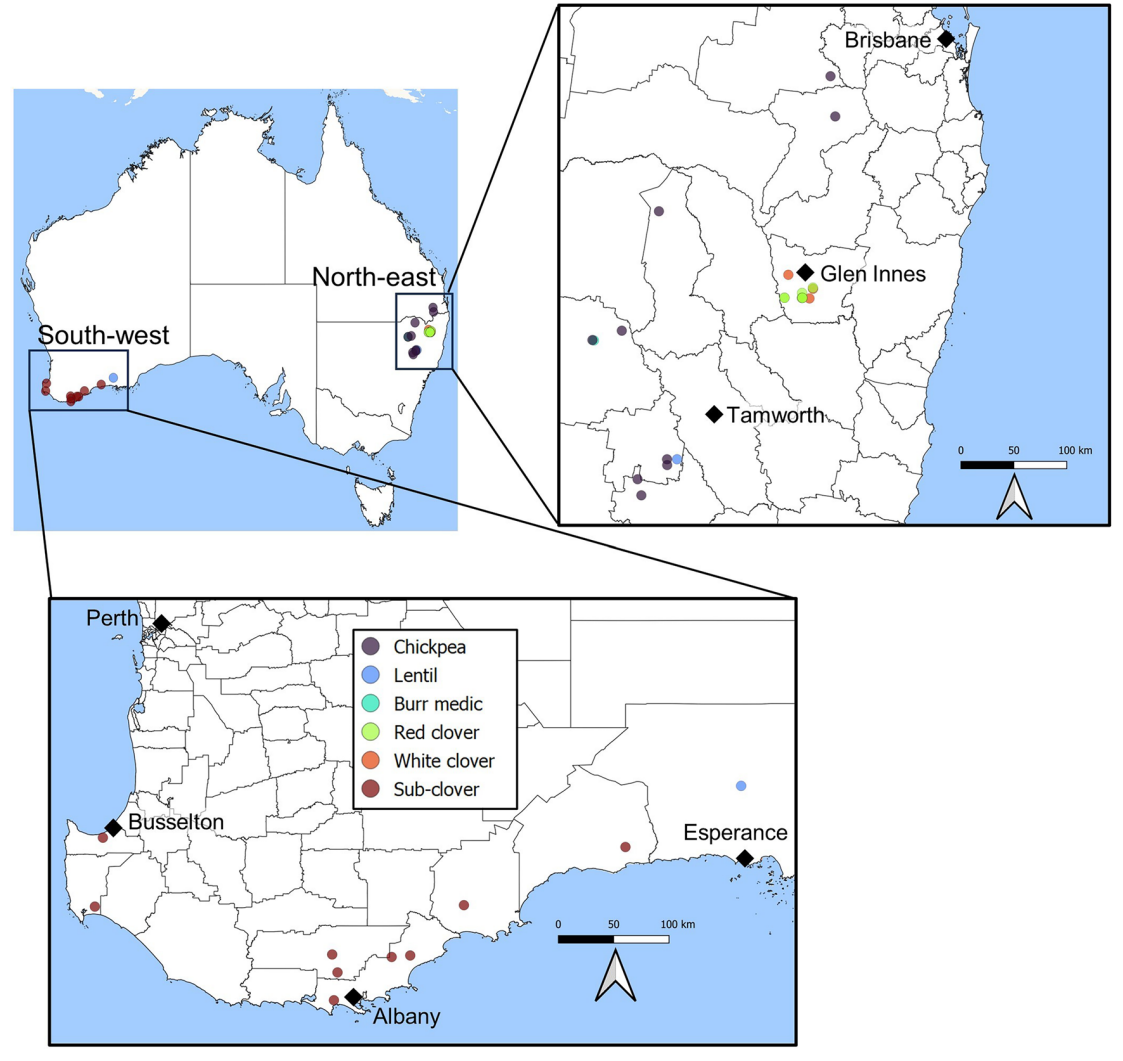
Locations and hosts of soybean dwarf virus isolates sequenced from the south-coast of south-west Western Australia (south-west) and north-east New South Wales/south-east Queensland (north-east) regions of Australia

The 41 new SbDV isolates sequenced in this study were collected from two geographically distinct production regions of Australia; the south coast of south-west WA (hereafter referred to as the ‘south-west’) and a ~45,000-km^2^ area of northern NSW/southern QLD (hereafter referred to as the ‘north-east’) (Fig. 1, Table 1). From the south-west, 10 isolates were collected, including nine from subterranean clover growing in dairy pastures on the south coast and one from lentil growing in Grass Patch in the Esperance region. From the north-east, 31 isolates were collected. These consisted of 10 each from red clover and white clover from mixed pastures growing within 30 km of Glen Innes. The remaining nine from chickpea, one from lentil, and one from burr medic (*Medicago polymorpha*) were from sites spanning from Pilton, Queensland, in the north to Breeza, NSW, in the south.

**Table 1:** Details of soybean dwarf virus isolates sequenced in this study

Accession number	Isolate	Source	Region^a^	Location	Year collected	Predicted clade^b^	Actual clade^c^
PP922787	5943	*Medicago polymorpha*	NE	Edgeroi, NSW	2013	-	DP
PP922781	5433	*Cicer arietinum*	NE	Croppa Creek, NSW	2014	-	DP
PP922786	5944	*C. arietinum*	NE	Spring Ridge, NSW	2015	-	DP
PP922782	5434	*C. arietinum*	NE	Breeza, NSW	2015	-	DP
PP922783	5436	*C. arietinum*	NE	Edgeroi, NSW	2015	-	DU
PP922784	5481	*C. arietinum*	NE	Colley Blue, NSW	2018	-	DP
PP922789	L43	*Trifolium repens*	NE	Lambs Valley, NSW	2022	Y	DP
PP922790	L45	*T. repens*	NE	Lambs Valley, NSW	2022	Y	DP
PP922791	L46	*T. pratense*	NE	Lambs Valley, NSW	2022	D	DP
PP922792	L51	*T. pratense*	NE	Glen Innes, NSW	2022	D	DP
PP922793	L56	*T. pratense*	NE	Matheson, NSW	2022	D	DP
PP922799	L59	*T. pratense*	NE	Matheson, NSW	2022	D	DP
PP922794	L60	*T. pratense*	NE	Shannon Vale, NSW	2022	D	DP
PP922795	L69	*T. pratense*	NE	Lambs Valley, NSW	2022	D	DP
PP922780	L71	*T. pratense*	NE	Lambs Valley, NSW	2022	D	DP
PP922796	L72	*T. repens*	NE	Lambs Valley, NSW	2022	Y	DP
PP922797	L77	*T. pratense*	NE	Lambs Valley, NSW	2022	D	DP
PP922798	L87	*T. pratense*	NE	Lambs Valley, NSW	2022	D	DP
PP922788	L42	*T. pratense*	NE	Lambs Valley, NSW	2022	D	DP
PP922785	5483	*C. arietinum*	NE	Breeza, NSW	2018	-	DP
PP922803	5435	*Lens culinaris*	NE	Breeza, NSW	2015	-	YP
PP922802	5432	*C. arietinum*	NE	Edgeroi, NSW	2013	-	YP
PP922811	L49	*T. repens*	NE	Glen Innes, NSW	2022	Y	YP
PP922812	L54	*T. repens*	NE	Reddestone, NSW	2022	Y	YP
PP922813	L61	*T. repens*	NE	Shannon Vale, NSW	2022	Y	YP
PP922814	L70	*T. repens*	NE	Lambs Valley, NSW	2022	Y	YP
PP922815	L76	*T. repens*	NE	Lambs Valley, NSW	2022	Y	YP
PP922816	L85	*T. repens*	NE	Lambs Valley, NSW	2022	Y	YP
PP922817	L86	*T. repens*	NE	Lambs Valley, NSW	2022	Y	YP
PP922809	5945	*C. arietinum*	NE	Pilton, QLD	2013	-	YP
PP922808	5946	*C. arietinum*	NE	Warwick, QLD	2013	-	YP
PP922804	6091	*T. subterraneum*	SW	Esperance, WA	2017	-	YP
PP922806	6694	*T. subterraneum*	SW	Narrikup, WA	2017	-	YP
PP922807	6931	*T. subterraneum*	SW	Gairdner, WA	2017	-	YP
PP922805	6692	*T. subterraneum*	SW	Mt Barker, WA	2017	-	YP
PP922800	BC2020	*L. culinaris*	SW	Grass Patch, WA	2019	-	YP
PP922801	3342	*T. subterraneum*	SW	Green Range, WA	2019	-	YP
PP922810	KF20	*T. subterraneum*	SW	Scott River, WA	2020	-	YP
PP922819	WA8	*T. subterraneum*	SW	Torbay, WA	2018	YP	YP
PP922820	McG	*T. subterraneum*	SW	Busselton, WA	2020	-	YP
PP922818	SS1	*T. subterraneum*	SW	South Stirlings, WA	2020	-	YP

^a^Production regions of Australia: south coast of south-west Western Australia (SW) and north-east New South Wales/south-east Queensland (NE)

^b^Based on available biological data – Y or D based on host range indicators (*T. repens, T. pratense*, *Phaseolus vulgaris, Lupinus albus*) and P or S based on primary vector species (*Acyrthosiphon pisum* or *Aulacorthum solani*, respectively)

^c^Based on clade in whole-genome nt sequence tree (Y or D) and N-terminal region of the readthrough domain aa sequence tree (P or S). U – undetermined

### RNA extraction and PCR confirmation

All RNA extractions were done on fresh or freeze-dried material using QIAshredder and RNeasy Mini Kits according to the manufacturer's instructions (QIAGEN, Germany). Two-step RT-PCR and Sanger sequencing were performed to confirm the presence of SbDV. Generic ‘*Luteoviridae’* primers AS2 (5’- ATCACBTTCGGGCCGWSTYTWTCAGA-3’) and AS3 (5’- CACGCGTCIACCTATTTIGGRTTITG -3’) were used to amplify a region of ORF3 [1]. To obtain cDNA, reverse transcription was performed using an ImProm-II Reverse Transcription System with random primers (Promega, USA). The cDNA was used to perform PCR amplification using GoTaq DNA polymerase (Promega, USA) with the reaction consisting of an initial incubation at 95°C for 1 min followed by 30 cycles of 95°C for 15 s, 50°C for 20 s, and 72°C for 60 s and a final extension at 72°C for 10 min. The product was analysed by 1% agarose gel electrophoresis to confirm bands and then purified using a QIAquick PCR Purification Kit according to the manufacturer's instructions (QIAGEN, Germany). The purified product was then sent to the Australian Genome Research Facility (AGRF) for Sanger sequencing. The resulting sequences were confirmed to be SbDV using the BLAST tool in Geneious Prime 2022.0.1 (Biomatters, New Zealand).

### RNA sequencing and genome sequence assembly

Total RNA of each isolate was sent to AGRF for plant ribosomal RNA depletion, library preparation, and barcoding before being sequenced on an Illumina NovaSeq instrument (Illumina, USA).

For each sample, reads were first trimmed using CLC Genomics Workbench (CLCGW) (formerly CLC Bio, Denmark, now QIAGEN, Germany) with the quality scores limit set to 0.01, the maximum number of ambiguities set to two, and removing any reads with <30 nucleotides (nt). Contigs were assembled using the *de novo* assembly function of CLCGW with automatic word size; automatic bubble size; minimum contig length, 500; mismatch cost, 2; insertion cost, 3; deletion cost, 3; length fraction, 0.5; and similarity fraction, 0.9. Contigs were sorted by length, and the longest was used as a query sequence for a BLAST search [2]. In addition, trimmed reads were imported into Geneious Prime 2022.0.1 and provided with a reference sequence obtained from the GenBank database (Table 2). Mapping was performed with a minimum overlap of 10%, a minimum overlap identity of 80%, "allow gaps" set to 10%, and fine-tuning set to iterate up to 10 times. The contig of interest from CLCGW and the consensus sequence from mapping in Geneious were used to create a consensus sequence in Geneious by alignment using Clustal W. ORFs were predicted and annotations were made using Geneious. Finalized sequences were submitted to GenBank (accession numbers PP922780-PP22820).

**Table 2:** Summary of sequencing data from 41 new soybean dwarf virus isolates

GenBank accession no.	Isolate ID	Number of reads	No. of reads after trimming	No. of contigs after assembly	Contig length (nt)	Reads mapped (*de novo*)	Average coverage (*de novo*)	Reference	Number of reads mapped to reference	Average coverage (mapped)	Final length (nt)
PP922804	6091	22,104,898	22,092,373	20,469	5,700	1,386,835	23,105	NC003056	1,452,334	22,638	5,609
PP922806	6694	20,628,914	20,613,554	20,115	5,926	207,565	3,408	n/a	n/a	n/a	5,609
PP922807	6931	22,607,850	22,592,006	31,839	5,887	1,702,978	27,895	n/a	n/a	n/a	5,609
PP922805	6692	19,395,142	19,383,697	21,324	5,878	1,314,610	21,560	n/a	n/a	n/a	5,609
PP922800	BC2020	53,522,120	53,522,098	25,478	5,831	1,173,236	19,004	n/a	n/a	n/a	5,604
PP922801	3342	39,230,352	38,334,678	20,459	5,917	689,614	11,447	n/a	n/a	n/a	5,609
PP922810	KF20	41,097,054	40,365,759	27,674	3,748; 1,142	1,041,052; 548,224	27,295; 46,052	LR584028	1,756,848	28,687	5,609
PP922819	WA8	57,384,178	55,951,527	30,772	5,950	552,795	9,146	n/a	n/a	n/a	5,609
PP922820	McG	53,079,454	51,851,854	25,577	6,037	3,479,926	56,778	n/a	n/a	n/a	5,611
PP922818	SS1	48,673,758	45,577,214	22,218	5933	3,519,284	58,145	n/a	n/a	n/a	5,609
PP922802	5432	62,076,358	60,037,094	32,357	5,108	2,608,915	50,176	n/a	n/a	n/a	5,609
PP922787	5943	52,993,582	51,377,805	30,309	4,908; 1,096	3,401,863; 871,626	66,628; 74,596	n/a	n/a	n/a	5,459
PP922809	5945	54,675,340	54,433,481	33,980	1,782; 1,202; 656; 737	1,928,006; 814,347; 146,719; 496,744	99,885; 65,075; 20,387; 58,227	n/a	n/a	n/a	5,697
PP922808	5946	42,405,138	42,129,029	25,414	5,876	2,265,159	38,413	n/a	n/a	n/a	5,701
PP922781	5433	46,109,438	45,908,319	22,653	5,190	5,680,097	108,557	n/a	n/a	n/a	5,459
PP922786	5944	57,146,620	56,927,262	19,071	5,706	712,689	12,448	n/a	n/a	n/a	5,459
PP922782	5434	47,162,646	46,922,166	23,475	5,756	5,187,565	89,779	n/a	n/a	n/a	5,459
PP922803	5435	53,056,370	52,900,341	27,969	2,214; 5,950	95,844; 1,317,816	4,214; 22,001	n/a	n/a	n/a	5,609
PP922783	5436	51,172,706	50,927,899	23,750	5,827	6,099,515	104,271	n/a	n/a	n/a	5,459
PP922784	5481	71,168,032	70,928,034	19,809	5,749	48,487,240	840,894	n/a	n/a	n/a	5,459
PP922785	5483	50,322,106	49,976,747	21,143	5,853	10,778,403	183,595	n/a	n/a	n/a	5,459
PP922788	L42	40,614,886	40,614,796	38,311	2966; 1723; 1091	624997; 308805; 117918	27301; 21971; 13950	MF627965	1,245,669	29,738	5,459
PP922789	L43	34,250,078	34,249,989	27,449	1235; 4298	954; 2943	101; 93	MF627965	4081	95	5,459
PP922790	L45	32,285,868	32,285,769	28,350	5,719	30,552	730	n/a	n/a	n/a	5,459
PP922791	L46	36,191,964	36,191,870	29,004	1415; 1388; 1094; 1163; 696	148405; 68970; 78667; 71214; 20253	14080; 6750; 9779; 8147; 3980	MF627965	551,306	13,051	5,459
PP922811	L49	32,620,208	32,620,129	30,069	2814; 735; 550; 927	135428; 24610; 23075; 279	6234; 4284; 5060; 39	LR584028	244,004	5,485	5,609
PP922792	L51	57,525,816	57,525,689	15,412	1271; 868; 1011; 1511; 715	18611; 3556; 12542; 24761; 10081	1819; 538; 1694; 2084; 1677	MF627965	85,570	1,988	5,459
PP922812	L54	36,607,670	36,607,571	54,297	3896; 951; 1032; 618	18901; 856; 3798; 3646	636; 114; 482; 754	LR584028	28,039	632	5,609
PP922793	L56	42,220,748	42,220,654	38,069	1827; 1119; 989; 772; 736; 764	153713; 86009; 23873; 22865; 7766; 42536	11449; 10175; 3207; 3663; 1332; 7555	MF627965	443,643	10,573	5,459
PP922799	L59	34,097,278	34,097,190	29,816	1541; 687; 879; 664; 638	42035; 15556; 12163; 20023; 16201	3388; 3014; 1692; 3989; 3269	MF627965	149,239	3,492	5,459
PP922794	L60	33,559,768	33,559,668	29,417	4445; 1078;1197	389872; 86205; 38284	11742; 10317; 3427	MF627965	520,441	12,346	5,459
PP922813	L61	51,478,278	51,478,160	49,745	5,852	344,489	8,006	NC003056	344,009	7,455	5,609
PP922795	L69	32,645,862	32,645,787	26,197	833; 845; 1083; 756; 546	23087; 47204; 78341; 53305; 28774	3680; 6762; 9650; 8625; 6739	MF627965	321,165	21,526	5,459
PP922814	L70	42,457,582	42,457,486	33,471	1471; 1651; 892	218610; 164822; 85476	18993; 12947; 12790	LR584028	657,403	15,227	5,609
PP922780	L71	37,889,610	37,889,513	27,161	1813; 686; 576; 620; 995; 571	143555; 34656; 39517; 6082; 3360; 4089	10244; 5222; 9180; 1119; 276; 602	MF627965	404,881	9,414	5,456
PP922796	L72	64,673,360	54,673,190	49,915	1600; 980; 2579; 1506; 1491; 1173	115905; 27536; 268243; 98190; 36396; 1548	9601; 3708; 14223; 8841; 3188; 170	MF627965	545,039	12,983	5,459
PP922815	L76	33,648,108	33,648,038	29,579	6,021	276,910	6,258	n/a	n/a	n/a	5,609
PP922797	L77	35,807,912	35,807,823	28,675	5,695	246,185	5,901	n/a	n/a	n/a	5,459
PP922816	L85	38,030,960	38,030,858	30,805	1729; 866; 932; 865; 701; 678	165881; 84164; 59107; 20979; 35343; 50424	12860; 12828; 7962; 3138; 6094; 9747	LR584028	513,228	11,861	5,609
PP922817	L86	38,375,122	38,375,022	31,052	680; 840; 715; 1316; 1012; 699	62832; 40110; 10896; 68542; 40892; 12155	11677; 5778; 1821; 6840; 5238; 2120	LR584028	291,276	6,936	5,609
PP922798	L87	28,236,728	28,236,652	21,438	2667; 853; 707; 675	101429; 11163; 2734; 18087	4844; 1686; 497; 3119	MF627965	177,534	4,090	5,459

### Phylogenetic analysis

All 50 available complete or near-complete genome sequences of SbDV, including three from Australia (Table 3), were downloaded from GenBank and aligned with the 41 new genome sequences from this study, using MAFFT [31]. The N-RTD sequence was extracted from the nucleotide sequence alignment and translated to an aa sequence alignment before analysis. Phylogenetic analysis was performed using the maximum-likelihood method and the HKY model with uniform rates for the nt alignment, and the maximum-likelihood method and the JTT matrix-based model for the aa alignment, both in MEGA X [34]. Pairwise nt % and aa % identity values were calculated in Geneious 2022.0.1, using the same alignments. Bean leafroll virus (accession number NC003369) was used as an outgroup for both trees.

**Table 3:** Soybean dwarf virus sequences obtained from GenBank and used in phylogenetic analysis

Accession number	Isolate	Host	Location	Year collected	Reference	Predicted clade^a^	Actual clade^b^
AB038150	M96-1 (DP)	Aphid	Japan	2001	[50]	DP	DP
MN412737	Kreis_Stormarn_16	*Pisum sativum*	Germany	2016	[16]	-	DP
MN412738	Kreis_Stormarn_18	*P. sativum*	Germany	2018	[16]	-	DP
MG600300	SDV-HZ3	*Trifolium pratense*	Czechia	2015	[35]	D	DP
MG600299	SDV-HZ1	*T. pratense*	Czechia	2015	[35]	D	DP
MF627965	HS128	*Vigna angularis*	Korea	2016	Unpublished	-	DP
OM953424	HS	*Glycine max*	Korea	2020	[24]	-	DP
MT526793	IA-2016	*G. max*	USA	2016	[13]	-	DP
MT526794	IA-2017	*G. max*	USA	2017	[13]	-	DP
MT669395	IA-2-2018	*G. max*	USA	2018	[13]	-	DP
MT669394	IA-1-2018	*G. max*	USA	2018	[13]	-	DP
KJ786321	C1IL2	*T. pratense*	USA	2009	[52]	D	DP
DQ145545	Wisc3	*G. max*	USA	2003	[9]	D	DP
KJ786322	W4	*G. max*	USA	2009	[52]	-	DP
OK030799	Market Weighton	*P. sativum*	UK	2019	[14]	-	DP
OR553429	MD2-D	*T. repens*	USA	1991	[47]	DP	DR
OR553431	MD3-D	*Chenopodium* spp.	USA	1991	[47]	DP	DP
OR553432	MD7-D	*G. max*	USA	1993	[47]	DP	DP
OR553433	MD8-D	*Medicago lupulina*	USA	1988	[47]	DP	DP
OR553434	MD9-D	*T. pratense*	USA	2005	[47]	DP	DP
OR553435	MD12-D	*T. incarnatum*	USA	2006	[47]	DP	DP
OR553439	NY-D	*T. pratense*	USA	1988	[47]	DP	DP
OR553440	PA-D	*T. hybridum*	USA	1988	[47]	DP	DP
OR553441	SC-D	*T. subterraneum*	USA	1991	[47]	DP	DP
OR553442	VA20-D	*T. subterraneum*	USA	1990	[47]	DP	DP
AB038149	HS97-8 (DS)	*G. max*	Japan	2001	[50]	DS	DS
AB076038	HS99-5 (DS)	*G. max*	Japan	1999	[51]	DS	DS
OR553424	Hok2-D	*G. max*	Japan	1981	[47]	DS	DS
LR584030	ESPCL2	*T. subterraneum*	Esperance, Aus	2013	[32]	-	YP
LR584029	ESPCL15-2	*T. subterraneum*	Esperance, Aus	2013	[32]	-	YP
AB038148	M94-1 (YP)	*G. max*	Japan	2001	[50]	YP	YP
MT543032	JKI ID 23556	*T. repens*	Germany	2007	[15]	YP	YP
MN412736	Muenster_16	*P. sativum*	Germany	2016	[16]	-	YP
JN674402	MD6-Y	*T. repens*	USA	2006	[53]	YP	YP
LR584028	NSWCP15-2	*Cicer arietinum*	NSW, Aus	2013	[32]	-	YP
OK030752	East Anglia	*P. sativum*	UK	2007	[14]	-	YP
OR553426	SY-Y	*Lens culinaris*	Syria	1994	[47]	YP	YP
OR553427	KY-Y	*T. repens*	USA	1990	[47]	YP	YP
OR553428	MD1-Y	*T. repens*	USA	1986	[47]	YP	YP
OR553430	MD2-Y	*T. repens*	USA	1991	[47]	YP	YP
OR553436	MD13-Y	*T. repens*	USA	2006	[47]	YP	YP
OR553437	MS-Y	*T. subterraneum*	USA	1989	[47]	YP	YP
OR553438	NC-Y	*T. repens*	USA	1990	[47]	YP	YP
OR553443	VA20-Y	*T. subterraneum*	USA	1990	[47]	YP	YP
OL472235	MIR20SW	Pooled weeds	Slovenia	2020	[43]	-	YP
AB038147	M93-1 (YS)	*G. max*	Japan	2001	[50]	YS	YS
L24049	Tas-1	*Vicia faba*	Tasmania, Aus	~1980s	[42]	YS	YS
OR553423	Hok1-Y	*G. max*	Japan	1981	[47]	YS	YS
OR553425	NZ-Y	*T. repens*	New Zealand	1986	[47]	YS	YS
LC663963	RG24	*G. max*	Japan	2017	[18]	-	YS
NC003369^c^	Bean leafroll virus	*V. faba*	USA	-	[10]	-	-

^a^Based on available relevant biological data – Y or D based on host range indicators (*T. repens, T. pratense*, *Phaseolus vulgaris*) and P or S based on vector species (*Acyrthosiphon pisum* or *Aulacorthum solani*)

^b^Based on clade in the whole-genome nt sequence tree (Y or D) and N-terminal region of the readthrough domain aa sequence tree (P or S), R -recombinant

^c^Used as outgroup in phylogenetic trees

## Results

### High-throughput sequencing

Across all 41 samples, the total number of reads after trimming for each sample ranged from 19,383,142 to 70,928,034. The sequences were assembled and/or mapped to a reference sequence, and the final genome sequences obtained were 5,511 nt to 5,752 nt in length, with an average coverage from 39 times to 840,894 times (across complete and partial *de novo-*assembled segments). The data for each sample, including any references used for mapping are shown in Table 2. In total, 41 SbDV genome sequences were obtained, all of which can be considered ‘near-complete’, containing the entire coding region and much of the 5' and 3' untranslated regions.

### Phylogenetic analysis of whole-genome nt sequences – D and Y clades

**Fig. 2:**
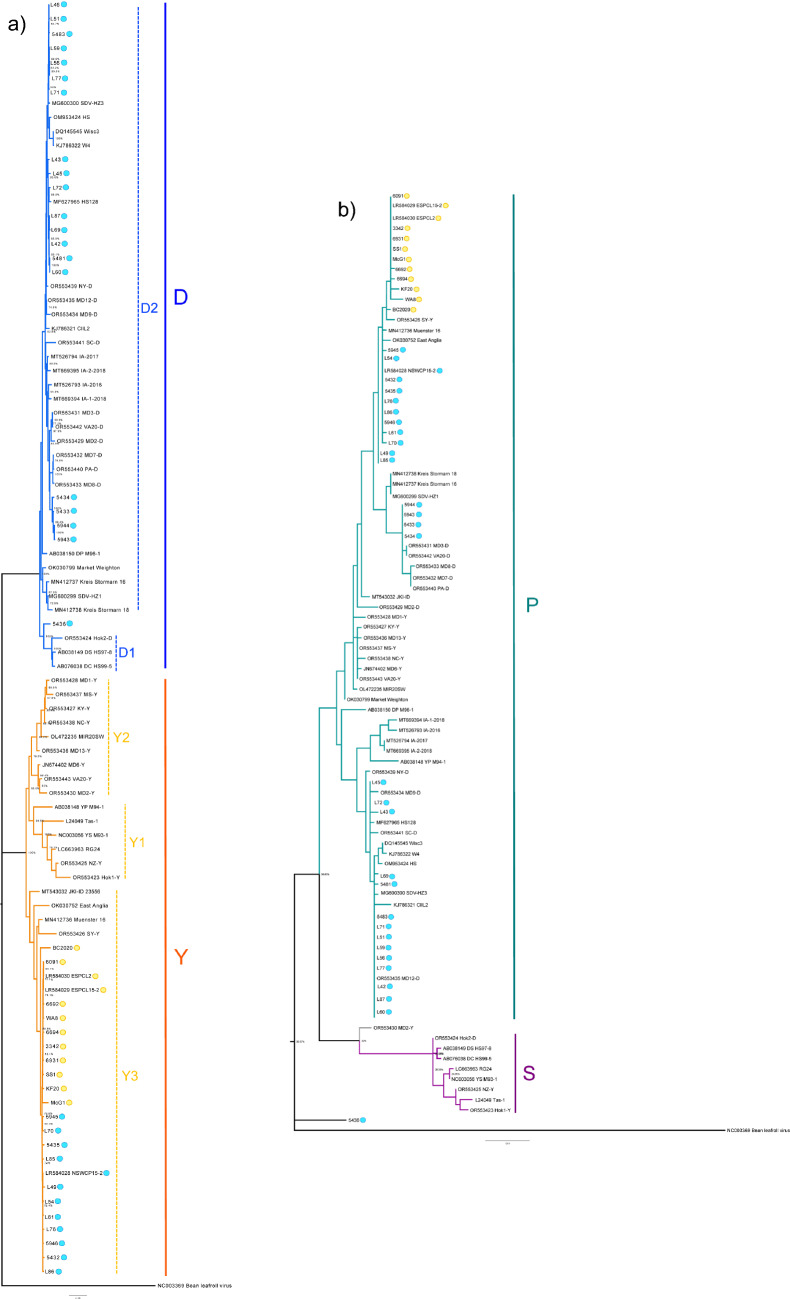
Mainland Australian isolates:  = north-east,  = south-west. Soybean dwarf virus phylogenetic tree of 92 whole-genome nucleotide sequences, including the reference sequence of bean leafroll virus used as an outgroup. The maximum-likelihood method and the Tamura-Nei model were used with 1000 bootstrap replicates. Annotations of D and Y subclades are as identified by Stone et al. [47]. (a) Phylogenetic tree of 92 partial amino acid sequences of the N-RTD region of soybean dwarf virus, including the reference sequence of bean leafroll virus as an outgroup. The maximum-likelihood method and the JTT matrix-based model were used with 1000 bootstrap replicates (b) Both trees shown here are the ones with the highest log likelihood. The percentage of trees in which the associated sequences clustered together is shown next to the branches.

When analysing the nt sequence of the whole genome, SbDV isolates separated into distinct D and Y clades with 77-80% nt sequence identity between them (Fig. 2a). Australian isolates were represented in both D and Y clades. Among the available isolates, there was more diversity within the Y clade than within the D clade. However, among Australian isolates, Y clade isolates were slightly less diverse (23 of 24 isolates had 99 to 100% nt sequence identity, and one had 95 to 97% nt sequence identity) than D clade isolates (95 to 100% nt sequence identity) despite having a broader geographical distribution.

The analysis supported the D and Y subclades identified by Stone et al. [47]. All mainland-Australian YP clade isolates originating from subterranean clover in the south-west and chickpea, lentil, and white clover in the north-east were highly similar, clustering in the Y3 subclade with isolates from Germany, the United Kingdom, and Syria. The 10 south-west isolates formed a tight cluster in the Y3 subclade with >95% nt sequence identity. Subterranean-clover-infecting isolates collected in 2013 from the south-west and sequenced in a previous study [32] had 99-100% nt sequence identity to isolates originating from subterranean clover growing 300-700 km to the west from 2017 to 2020. In the north-east, all 11 Y clade isolates sequenced in this study and a chickpea-infecting isolate collected in 2013 in a previous study [32] had 99% nt sequence identity and also fell into the Y3 subclade. These grouped with the south-west Y clade isolates, mostly with 99% nt sequence identity, except for two south-west isolates: McG1 (97 to 98% nt sequence identity) from Busselton, with variation concentrated in ORF1, and BC2020 (96 to 97% nt sequence identity) from Grass Patch, with variation concentrated in the 3’ half of ORF5 (a variable region of the SbDV genome). These isolates had 95% nt sequence identity to each other. The only other Y clade isolate sequence from Australia was Tas-1, which fell in the Y1 subclade with isolates from New Zealand and Japan.

The 20 D clade isolates from the north-east had ~80% nt sequence identity to Y clade isolates from the same region, and sometimes from the same pasture sward (e.g., isolates L45 and L70). Among the D clade isolates, there was 95 to 100% identity, with 19 isolates falling into the D2 subclade with isolates from the eastern USA, Korea, the Czech Republic, Germany, the United Kingdom, and Japan. The most divergent isolate, 5436 from chickpea, fell outside the D1 and D2 subclades, with 96% nt sequence identity to both D1 and D2 subclade isolates. The D clade isolates infecting red clover swards in 2022 were 97 to 100% identical to all other chickpea-infecting isolates and the one medic-infecting isolate collected 200 to 300 km to the south and west between 2013 and 2018. None of the south-west isolates fell into the D clade.

### Phylogenetic analysis of N-RTD aa sequences – P and S clades

When analysing the N-RTD aa sequence, 81 out of 91 sequences grouped in the P clade, eight grouped in the S clade, and two fell outside the two clades (5436 and MD2-Y) (Fig. 2b). Isolate Tas-1, originating from Tasmania and transmitted by *Au. solani*, fell into the S strain clade. The other 44 Australian isolates fell into the P strain clade, including all of those sequenced in this study. Isolate 5436, which fell outside the P and S strain clades, had 89-93% aa sequence identity to the P clade isolates, 83-87% aa sequence identity to the S clade isolates, and 89% aa sequence identity to the recombinant isolate MD2-Y. Although the aa sequence from isolate 5436 had many unique aa substitutions, it had P-type residues at 11 of the 12 positions (E97 being the exception) identified by Stone et al [47] as potential determinants of vector specificity.

## Discussion

In this study, we conducted phylogenetic analysis of the near-complete genome nt sequences and the N-RTD aa sequences of 44 SbDV isolates from mixed-cropping regions in the south-west and north-east of mainland Australia (41 newly sequenced in this study) together with 46 isolates from nine other countries. At the whole-genome level, the isolates separated into D and Y clades. At the N-RTD level, most of the isolates separated into P and S clades. All of the south-west isolates and 11 of the 31 north-east isolates were in the Y clade, and the remaining 20 north-east isolates were in the D clade. Except for one isolate that fell outside the P and S clades, all south-west and north-east isolates were in the P group. Host range and/or vector species data available for 34 of 50 isolates obtained from GenBank and 21 of 41 isolates from this study supported the inferences from phylogenetic analysis, except for three D clade isolates sequenced in this study that originated from white clover in a mixed red and white clover sward in the north-east. These analyses suggest that the YP strain is predominant in the south-west and YP and DP strains are predominant in the northeast, suggesting that *Ac. pisum*, *M. persicae*, and possibly *Ap. craccivora* are the key SbDV vectors in these regions and thus targets for effective virus management. The Australian SbDV phylogeny is an important resource for future research and will facilitate the development of robust strain-specific diagnostic assays.

The genetic similarity of south-west isolates collected over the past decade suggest that the YP strain and its vectors are involved in the repeated epidemics of leaf reddening disease in subterranean clover in the south-west [44]. This inference is supported by phenotypic data for one of these isolates (WA-8), which was transmitted by *Ac. pisum* and *M. persicae* and able to infect white clover, common bean, and albus lupin, but not red clover [6], as well as the known prevalence of SbDV in white clover pastures in this region [37, 38]. In the north-east, both DP and YP are implicated in disease of chickpea [46] and probably other grain legumes. Y and D isolates collected from white and red clover swards had a high degree of nt sequence similarity to isolates in the same clade collected from grain legumes ~250 km to the north, south, and west from 2013 to 2018 (99% and 95-100% for Y and D clade isolates, respectively). This suggests an epidemiological link between grain and pasture legume production across the region – i.e., widespread SbDV infection in perennial pasture/weed species such as white and red clover could be providing a sustained reservoir of both SbDV and its vectors for spread into sensitive grains crops. Given the prevalence of P strain isolates, the risk of an epidemic in both regions analysed is likely to be determined by the population growth and movement of P strain vectors between pastures/weeds and crops, both with the potential to play the role of virus/vector source. This information will enable management strategies that target these potentially crucial aspects of SbDV.

The genetic and biological data together suggest that at least three of the four SbDV strains are present in Australia (YP, YS, and DP) but have differing geographical distributions. The high similarity between YP strain isolates collected in the south-west and north-east likely reflects recent and related incursions of this strain into these regions. *Ac. pisum* was first identified on mainland Australia in Victoria in 1980, and it had spread throughout NSW by 1982 and was being reported in WA by the late 1980s [5, 11]. SbDV infection of subterranean clover was reported in both regions as early as 1984, but the strain responsible could not be deduced [20]. At that time, both *M. persicae* and *Ap. craccivora* had been distributed across Australia for at least several decades [12] and thus could have also introduced SbDV YP or DP into these regions. No DP isolates were detected in the south-west, which could be explained by the absence of significant red clover cultivation in the region. No S clade isolates were found in the south-west or north-east. *Au. solani* was responsible for the first reported SbDV outbreaks on mainland Australia, in Victoria in the mid-1960s, and was also present in NSW and QLD by the mid-1960s [12]. Therefore, it is probable that S strains are present in NSW and QLD in SbDV-susceptible crops, which *Au. solani* frequently colonises. *Au. solani* has been present in the south-west region for at least several decades [5] but is uncommon in the host species studied here. The geographical barrier of the Nullarbor Plain likely also plays a major part in the apparently narrower genetic diversity of SbDV in the south-west. Future work should involve sequencing isolates collected from the south-east of mainland Australia (south NSW and Victoria), including any archived isolates available from the early outbreaks in subterranean clover, to get a more comprehensive picture of SbDV diversity in grain and pasture legumes grown in Australia. Furthermore, surveillance studies could provide information about the prevalence and diversity of SbDV in horticultural areas across Australia and any links that exist to isolates found in broadacre production.

Over 90% of SbDV isolates now available in the GenBank database are P clade isolates, including all isolates from the United Kingdom, mainland Europe, mainland Australia, the USA, and Syria, whilst just eight S strain isolate sequences are available, and they are limited to Japan (including all DS isolates sequenced), Tasmania, and New Zealand, in which they are reported to be most common [3, 27, 55]. This mainly reflects the fact that the largest sequencing studies have been done in regions where S strains are uncommon or absent – i.e., a study involving sequencing of a comparable number of isolates from soybean in Japan or vegetable legumes in New Zealand would be expected to change the P/S sequence proportion. However, it is also apparent that S strains have a smaller vector range [26] and, by extension, a smaller effective host range, which contributes to their absence in many of the regions studied.

Three north-east D clade isolates (L43, L45, and L72) were found in white clover growing in a mixed red and white clover sward, providing supporting evidence that white clover is not a strict Y strain indicator host [45]. Schneider et al. [45] also found that Y isolates can infect red clover plants when coinfected with a D strain isolate. However, in other cases, red clover was completely resistant to Y isolates [6, 28], and no Y isolates were found in red clover in this study. Although no mixed infection of strains was detected in our study, mixed pasture swards would facilitate mixed infections and provide an opportunity for SbDV recombinants with unique phenotypes to emerge. Furthermore, there is evidence that vector specificity is not always strict. Ashby et al. [4] reported that an S isolate from New Zealand could be transmitted with poor efficiency by *Ac. pisum*, and Schneider et al. [45] found that a mixed infection of a YP and a DP isolate was transmitted inefficiently by *Au. solani.* Of all of the isolates, isolate 5436 was the most diverse globally in the N-RTD and fell outside the P and S clades, but it resembled the P type at most of the important residues that are potential determinants of vector specificity [47]. It is plausible that this variation may influence this isolate’s transmissibility and vector species range. More-comprehensive host and vector range studies of Australian isolates, especially those involving recombinants, unique isolates, and mixed infections, would allow the inferences made in this study to be tested and broaden our understanding of SbDV biology and its genetic influences.

SbDV isolates also vary in their virulence, i.e., the severity of disease caused. Helms et al. [19] examined the virulence of three *Au. solani*-transmitted isolates (NSW-B, NSW-K, and Tas-1) on subterranean clover and found that NSW-K was significantly more virulent. However, sequence data are available only for Tas-1. Just one other sequenced Australian isolate (WA-8) has been phenotyped for virulence, and it caused severe disease in subterranean clover, chickpea, lentil, faba bean, and field pea [6]. Damsteegt [7] demonstrated that a DS isolate and a YS isolate differed in their transmissibility, symptomatology, and virulence across different hosts. Stone et al. [47] found that Y isolates that cause severe disease in soybean clustered strongly in a phylogenetic tree based on the movement protein (ORF4), indicating that it could be a determinant of virulence. Comparing the virulence of genetically different isolates on key hosts such as subterranean clover and grain legumes would help to identify any genomic determinants of this important trait.

This study used established relationships between phylogenetic clades and phenotypes to infer biological information from analysis of plant virus sequence data. In recent years, the warranted enthusiasm around new diagnostic and genome sequencing technologies has come at the cost of generating accompanying biological data [23]. Now that genome sequencing is an established tool in plant virology, a renewed focus on phenotyping genetic variants is likely to provide transformative meaning and value to the data generated by sequencing.

## Supplementary Information

Below is the link to the electronic supplementary material.

Supplementary file1 (FASTA 222 KB)

Supplementary file2 (TXT 532 KB)

## Data Availability

The datasets generated and/or analysed in the current study are available from the corresponding author on reasonable request.
